# Simulation Methods for MEMS S&A Devices for 2D Fuze Overload Loading

**DOI:** 10.3390/mi14081566

**Published:** 2023-08-07

**Authors:** Zhibo Wu, Yanbing Zhang, Chuanmeng Sun, Lei Feng, Shuangfeng Liu, Bin Jiao

**Affiliations:** 1 School of Electrical and Control Engineering, North University of China, Taiyuan 030051, China; zhybg_1126@nuc.edu.cn (Y.Z.); suncm@nuc.edu.cn (C.S.); fenglei@nuc.edu.cn (L.F.); liusf@nuc.edu.cn (S.L.); jiaobin1995@163.com (B.J.); 2 State Key Laboratory of Dynamic Measurement Technology, North University of China, Taiyuan 030051, China

**Keywords:** fuze MEMS S&A devices, recoil overload, centrifugal overload, experimental test

## Abstract

An experimental testing system for the two-dimensional (2D) fuze overload loading process was designed to address the loading issues of recoil overload and centrifugal overload in fuze safety and arming (S&A) device. By incorporating centrifuge rotation energy storage, impact acceleration simulation, and equivalent centrifugal rotation simulation, a block equipped with a fuze S&A device accelerated instantly upon having impact from a centrifuge-driven impact hammer, simulating recoil overload loading. The impact hammer was retracted instantaneously by adopting an electromagnetic brake, which resulted in the centrifugal rotation of the block around its track, to simulate the centrifugal overload loading. The dynamic equations of the experimental testing system and the equations of impact hammer motions were established, whereby the rotation speed of the centrifuge and the braking force of the electromagnetic brake were calculated and selected. A dynamic model of the collision between the impact hammer and block was established using ANSYS/LS-DYNA software for simulation analysis. The acceleration curves of the recoil overload and centrifugal overload with variations in the centrifuge speed, cushion material, and buffer thickness were obtained, which verified the feasibility of the proposed loading simulation method. Two-dimensional overload loading simulation tests were performed using the developed experimental testing system, and the acceleration curves of the recoil overload and centrifugal overload were measured. The test results indicated that the proposed system can accomplish 2D overload loading simulations for a recoil overload of several 10,000× *g* and centrifugal overload of several 1000× *g*.

## 1. Introduction

Fuze is the ultimate actuator for weapon systems to exert terminal damage effects, and its safety and reliability directly determine the success or failure of weapons [1,2,3,4]. An essential field of microelectromechanical systems (MEMS)’s application in fuze is MEMS safety and arming (S&A) devices [5,6,7,8,9]. MEMS S&A devices are significant components of fuze, and their sensitivity to the environment determines the reliability of the fuze function [10,11]. During the launching of shells, MEMS S&A devices are continuously loaded in two-dimensional (2D) overload conditions with high-impact recoil overload and high-centrifugation rotation overload [12,13,14]. If an abnormal overload occurs, the arming process may occur too early, resulting in dangerous conditions and possible misfire [15,16,17]. In order to test the performance of the fuze MEMS S&A devices under laboratory conditions and ensure their reliability, it is necessary to investigate simulation methods for the continuous 2D loading process with recoil high-impact overload and rotation high-centrifugation overload [18,19].

Peng Wang [20] designed an experimental testing system for 2D overload loading on a MEMS S&A device, in which the acceleration magnitude and loading time can be adjusted within a certain range by changing the motor speed and torque of the torsion spring. Hongjun Xiang [21] designed a centrifugal testing machine that simulates the dual environmental forces during fuze launching by installing a motor on the horizontal arm in the rotation shaft to enable its autorotation. Junli Zhao [22] and Weijun Zhong [23] investigated the internal ballistics of a gas gun. They simulated the dynamic overload conditions of fuze by adjusting the chamber pressure during the firing process of the gas gun. Yanbing Zhang [24,25,26] designed a compound centrifuge with a vector turntable started by the boosting of the gas gun. In their design, the centrifuge is initiated instantly by launching the gas gun to boost the rotation arm, and the vector turntable is driven by a servomotor simultaneously; thus, the launching and maneuvering flight processes of missiles are simulated. Using the impact–rotation method, Qingxi Yang [27] investigated the firing approach for arming fuze, established a collision model of instant and inertial firing, simulated the collision firing process, and conducted a performance test of fuze firing by adopting this method. Haiying Qian [28] and Man Xu [29] adopted the impact–rotation method to investigate the relationship between the performance parameters of fuze liquid storage batteries with respect to the recoil force and rotation speed. Chen Lin [30] designed a test set involving a multi-stage induction coilgun with a rotary tube to simulate the dual conditions of recoil and rotation during fuze launching.

As indicated by the literature, the current experimental testing systems not only suffer from complex configurations, large volumes, and high costs, but also can only perform two-dimensional overload with a small magnitude or single overload with a large magnitude. With a focus on a MEMS S&A device of a 20 mm small-caliber projectile, the objective of this study was to develop a method for simulating the continuous 2D fuze overload loading. The aim of this study is to explore the methods for evaluating the reliability performance of fuze MEMS S&A devices and their key components under laboratory conditions. Further, we tried to design an experimental testing system and use it to verify the feasibility of the simulation methods. The innovation of this study is to use a centrifuge to drive an impact hammer to have impact on the block equipped with the fuze MEMS S&A device at a high speed and accelerate it instantly. The impact acceleration method can be used to simulate higher recoil overload, simulating centrifugal overload by using the block to rotate around a circular track instead of a projectile rotating at high speed. The advantage of this method is that by increasing the centrifugal radius, the rotational speed is reduced, which will be easier to achieve compared to existing experimental test methods.

## 2. MEMS S&A Device

### 2.1. Basic Composition of MEMS S&A Device

**Figure 1:**
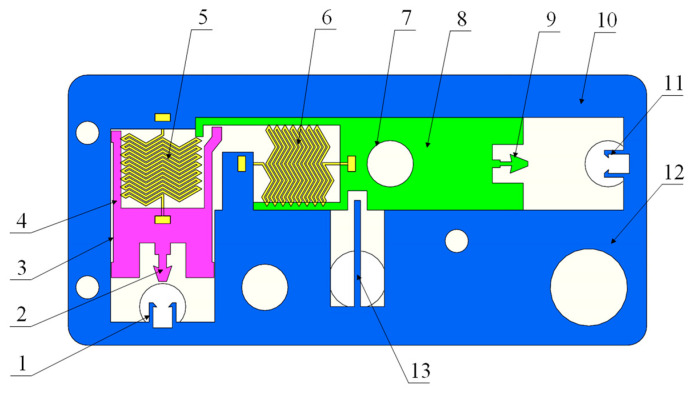
Schematic of the MEMS S&A device for 20 mm small-caliber projectiles. 1. Clamping holder of recoil safety unit, 2. clamping head of recoil slider, 3. Z-type groove, 4. recoil slider, 5. spring of recoil safety unit, 6. spring of centrifugal safety unit, 7. flash hole, 8. centrifugal explosion-proof slider, 9. clamping head of centrifugal explosion-proof slider, 10. substrate beam, 11. clamping holder of centrifugal safety unit, 12. substrate, 13. positioning safety lever.

The basic composition of a MEMS S&A device for 20 mm small-caliber projectiles is illustrated in Figure 1. It adopts a vertical substrate structure, with a maximum thickness of less than 1 mm, and is processed from UV-LIGA nickel material. According to the functions of the components, it can be divided into a recoil protection releasing unit and a centrifugal protection releasing unit [31]. The former utilizes the recoil force from projectile launching to release the movement constraints on the centrifugal explosion-proof slider, to release the recoil protection of the MEMS S&A devices. The latter adopt the rated centrifugal force from the projectile rotation to relieve the isolation effect of the centrifugal explosion-proof slider on the detonation sequences, thereby releasing all the protections in the system.

### 2.2. Environmental Forces in Chamber of MEMS S&A Device

**Figure 2:**
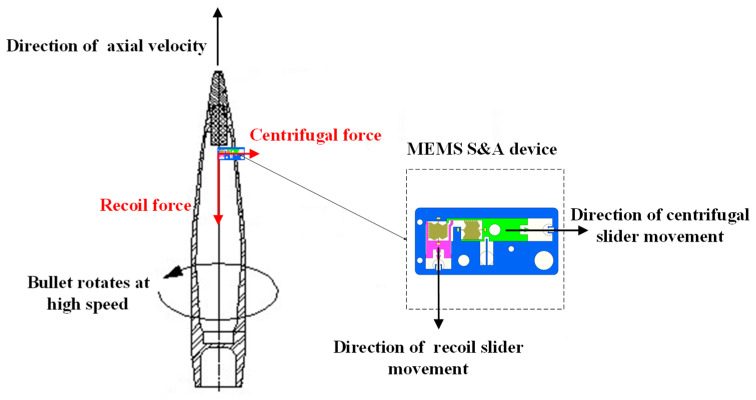
Forces and movements of MEMS S&A device in the chamber.

During the launch process, the axial velocity of the projectile rapidly increases to hundreds of meters per second under the chamber gas pressure generated by propellant combustion. Meanwhile, the MEMS S&A device is subjected to considerable launch recoil. The recoil slider overcomes the spring tension and moves downward. When it reaches the bottom, the clamping head on the recoil slider enters the clamping holder on the recoil safety unit and is constrained from resetting by the holder, releasing the first protection of the system (recoil protection). Due to the rifling in the barrel, the rotational speed of the projectile squeezed into the barrel rapidly increases to several 1000 or even 10,000 rotations per minute (r/min). Accordingly, the MEMS S&A device is also subjected to considerable centrifugal force. Under the centrifugal force, the centrifugal explosion-proof slider overcomes the spring tension and moves outward, and it is then fixed by the clamping holder on the centrifugal protection unit. The explosion-proof effects on the detonation sequence are then relieved, completely releasing the protections of the system (centrifugal protection). The forces and movements of the functional units in a MEMS S&A device are presented in Figure 2.

## 3. Simulation Method for 2D Overload Loading

### 3.1. Principle of Equivalent Similarity Simulation

**Figure 3:**
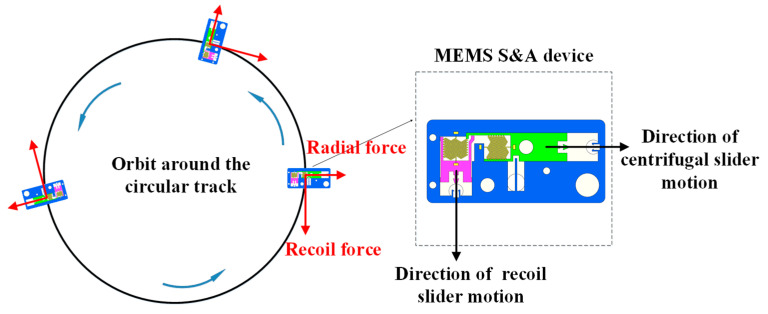
Schematic of the equivalent centrifugal rotation simulation.

According to the previous description, during projectile launching, the recoil slider in the MEMS S&A device moves downward along the projectile axis, and the centrifugal explosion-proof slider moves outward along the radial direction. A reference object is established by adopting the substrate of the MEMS S&A device. According to the reference, the recoil slider is constantly subjected to a downward force, while the centrifugal explosion-proof slider is constantly subjected to an outward lateral force. Therefore, according to the principle of equivalent similarity, the recoil environmental force during projectile launching can be simulated using the impact acceleration method, and the lateral force on the centrifugal explosion-proof slider can be simulated using equivalent centrifugal rotation (because the rotation speed of projectiles can reach several 10,000 r/min and because of the limitations of dynamic balance factors and motor speed, it is difficult to directly simulate projectile autorotation). The advantage of this method is that the rotation speed can be reduced by increasing the centrifugal radius. A schematic of the equivalent centrifugal rotation simulation is shown in Figure 3.

### 3.2. Experimental Testing System

**Figure 4:**
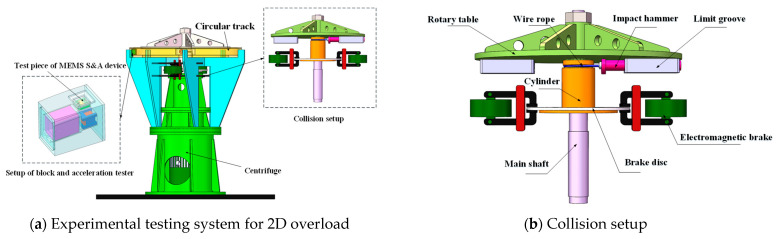
Structure of the experimental testing system.

As shown in Figure 4a, the experimental testing system for 2D continuous overload loading mainly includes a centrifuge, a collision setup, a circular track, and an acceleration tester. The centrifuge is composed of a rotary table, a main shaft, and a motor with variable frequency and adjustable speed, as well as a bench. The collision setup is installed beneath the rotary table, and the latter is located in the same horizontal plane as the circular track. The block, which is equipped with a MEMS S&A device and an acceleration tester, is placed in a circular track and remains stationary.

As shown in Figure 4b, the collision setup is the core component of the experimental testing system, mainly including a limit groove, an impact hammer, wire rope, a cylinder, a brake disc, and an electromagnetic brake. The limit groove is fixed beneath the centrifugal rotary table, while the impact hammer is inserted into the limit groove and can perform reciprocating telescopic motions. The wire rope is wound around the cylinder with one end fixed on the cylinder and the other connected to the impact hammer. The cylinder and rotary table are assembled coaxially and matched via a tooth–groove structure, allowing relative rotation within a certain angular range. The brake disc is fixed at the bottom of the cylinder and two electromagnetic brakes are installed symmetrically on the two sides of the brake disc with independent controlling functions.

### 3.3. Working Process of Experimental Testing System

#### 3.3.1. Energy-Storage Process in Centrifuge

**Figure 5:**
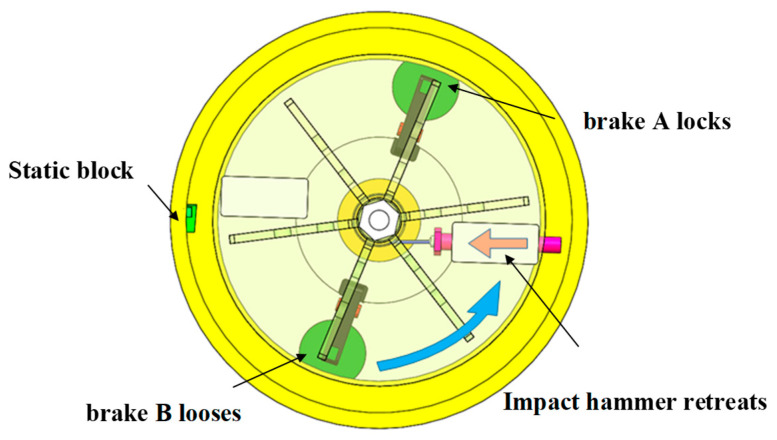
Energy-storage process in the centrifuge.

Before testing, the block equipped with the test piece of the MEMS S&A device is placed in the circular track and remains stationary with electromagnetic brake A locking the brake disc and electromagnetic brake B remaining loose. The variable-frequency motor is started and drives the centrifugal rotary table via the main shaft. Owing to the locking effect on the brake disc fixed with the cylinder, the cylinder is inhibited from rotating and remains stationary. Therefore, relative rotation occurs between the cylinder and the centrifugal rotary table. As the wire rope winds around the cylinder, the impact hammer is pulled back to the bottom of the limit groove. As shown in Figure 5, after the rotary table turns to a certain degree, its rotation becomes limited. As the torque output of the motor increases constantly, the rotary table and the cylinder overcome the friction braking force imposed by electromagnetic brake A (the torque output of the motor becomes larger than the braking torque of electromagnetic brake A), and then the rotary table rotates together with the cylinder and the braking disc (the static friction of electromagnetic brake A changes to dynamic friction) until the preset rotation speed is reached.

#### 3.3.2. Recoil Overload Loading Process

**Figure 6:**
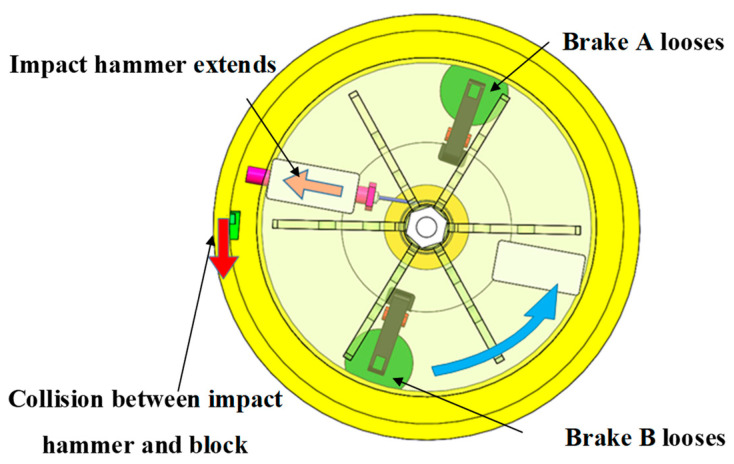
Recoil overload loading process.

After the motor reaches its preset speed and remains stable, the recoil overload loading process is initiated. When the photoelectric sensor detects that the impact hammer has rotated to a preset position, it releases electromagnetic brake A and deactivates the friction torque. Under the effects of centrifugal force, the impact hammer is spun out along the limit groove with its head extending into the circular track. As shown in Figure 6, the impact hammer collides with the block equipped with the MEMS S&A device at a high speed, simulating the recoil overload loading process.

#### 3.3.3. Centrifugal Overload Loading Process

**Figure 7:**
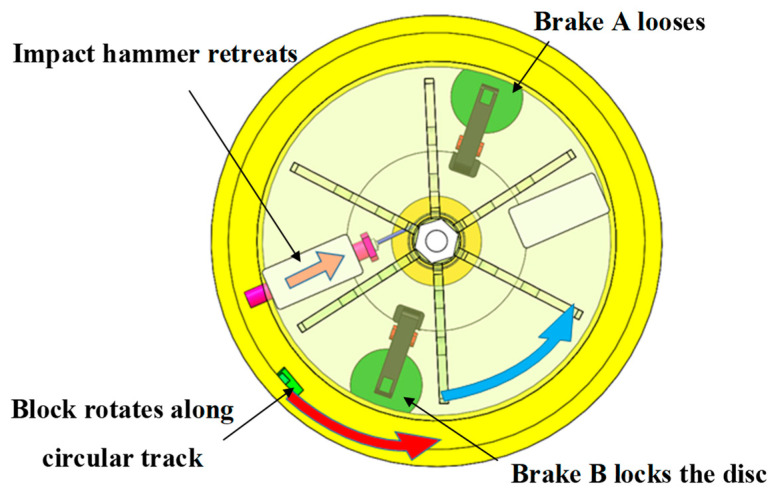
Centrifugal overload loading process.

Under the high-speed collision of the impact hammer, the block acquires a certain speed and performs inertial motion along the circular track. Meanwhile, as the block is subjected to a centrifugal force, centrifugal overload loading is performed. The block speed after collision is higher than the speed of the impact hammer, leading to a secondary collision between the block and the impact hammer. To avoid this situation, electromagnetic brake B has been added to the system. As shown in Figure 7, after the collision between the impact hammer and the block, electromagnetic brake B locks the brake disc to provide the cylinder with a braking torque. Meanwhile, as the wire rope winds around the cylinder, the impact hammer retreats to the bottom of the limit groove under the pulling force of the wire rope.

## 4. Theoretical Calculations

### 4.1. Criteria for 2D Overload

Dynamically simulating recoil overload conditions during fuze launching requires a recoil overload acceleration (*A*_recoil_) of >10,000× *g* and an overload duration of ≥500 μs. Dynamically simulating the centrifugal overload conditions generated by the high-speed rotation of the projectile under the effects of rifling during launching requires a centrifugal overload acceleration (*A*_centrifugal_) of >1000× *g*.

### 4.2. Centrifuge Speed

The recoil overload is enabled by the high-speed collision between the impact hammer and the block, and its peak load and pulse width are associated with the centrifuge speed. The high speed of the centrifuge results in a large amount of kinetic energy in the system and the contact time during the collision is short (~500 μs), assuming the conservation of kinetic energy and momentum in the system. Therefore, the angular velocity of the rotary table before and after the collision can be expressed as follows:

(1) $$\{\begin{array}{l} \frac{1}{2}J\omega_{0}{}^{2}=\frac{1}{2}J\omega_{1}{}^{2}+\frac{1}{2}mv_{1}{}^{2}\\ J\omega_{1}+m_{\mathrm{block}}\omega_{1}r_{\mathrm{block}}=J\omega_{0} \end{array}$$

where *J* = 43.2 kg·m^2^ represents the rotational inertia of the centrifuge system, *m*_block_ = 0.78 kg represents the mass of the block, *r*_block_ *=* 0.68 m represents the rotational radius of the block, ***ω***_0_ represents the rotational angular velocity of the centrifuge before collision, ***ω***_1_ represents the rotational angular velocity of the centrifuge after collision, *v*_0_ represents the block velocity before collision, and *v*_1_ represents the block velocity after collision.

According to (1), the angular velocity of the centrifuge is expressed as:

(2) $$\omega_{0}=\frac{m_{\mathrm{block}}v_{1}r_{\mathrm{block}}{}^{2}+Jv_{1}}{2Jr}$$

Assuming that the recoil overload has an ideal half-sinusoid waveform, the maximum block velocity (*v*_max_) after collision is expressed as:

(3) $$\{\begin{array}{c} \bar{A}=\frac{\omega }{\pi }\int_{0}^{\frac{\pi }{\omega }}A_{\mathrm{recoil}}\sin\omega tdt=\frac{2}{\pi }A_{\mathrm{recoil}}\\ v_{\max}=\int_{0}^{\tau }\bar{A}dt=\frac{2}{\pi }A_{\mathrm{recoil}}\tau \end{array}$$

Calculations yield ***ω***_0_ = 23.12 rad/s.

Therefore, to ensure that the acceleration magnitude and pulse duration of the recoil overload reach 10,000× *g* and 500 μs, respectively, the corresponding centrifuge speed is 221 r/min.

The centrifugal overload acceleration is related to the rotational speed of the block along the circular track. The centrifuge acceleration of the block (*A*) is expressed as:

(4) $$A_{\mathrm{centrifugal}}=\frac{v_{2}{}^{2}}{r_{\mathrm{block}}}$$

Calculations yield *v*_2_ = 82.46 m/s. By introducing *v*_2_ into (2), ***ω***_0_ = 61.14 rad/s is acquired.

Therefore, to ensure that the acceleration magnitude of the centrifugal overload reaches 1000× *g*, the corresponding centrifuge speed is 584 r/min.

In engineering practice, the waveform of the shock pulse is typically adjusted by adding a cushion. The pulse width of recoil overload can be calculated as follows [32]:

(5) $$\{\begin{array}{c} T=\pi \sqrt{\frac{mM}{K(m+M)}}\\ K=\frac{ES}{l} \end{array}$$

where *T* represents the duration of shock pulse, *m* represents the mass of the block with a cushion, *M* represents the equivalent mass of the centrifuge system, *K* is the stiffness coefficient of the system, *E* represents the elastic modulus of the cushion material, *S* represents the area of the cushion, and *l* represents the thickness of the cushion.

In summary, by setting the centrifuge speed in the range of 600–900 r/min, the requirements for the loading simulation of both recoil overload and centrifugal overload can be satisfied simultaneously.

### 4.3. Braking Forces

**Figure 8:**
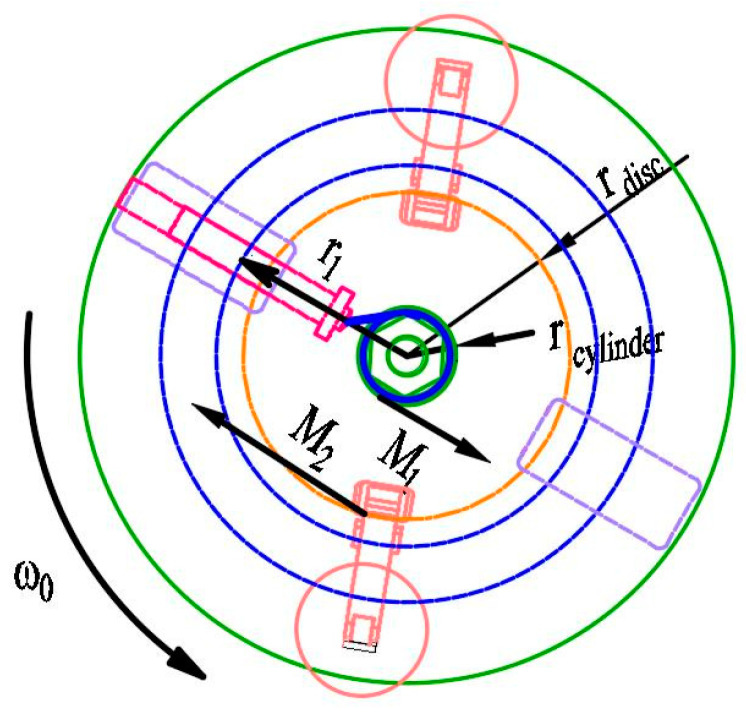
Force analysis of the impact hammer.

To ascertain the braking force of the electromagnetic brakes, it is necessary to analyze the forces imposed on the impact hammer (Figure 8). The centrifugal force on the impact hammer is expressed as:

(6) $$F_{1}=m_{\mathrm{hammer}}\omega^{2}r_{1}$$

where *m*_hammer_ = 1.5 kg represents the mass of the impact hammer, ***ω*** represents the rotational angular velocity of the impact hammer, and *r*_1_ = 0.35 m represents the rotational radius of the impact hammer.

From (6), the centrifugal speed is calculated as 700 r/min, and the centrifugal force on the impact hammer is 2821 N.

The torque from the centrifugal force of the impact hammer on the cylinder is expressed as:

(7) $$M_{1}=F_{1}r_{\mathrm{cylinder}}$$

where *r*_cylinder_ = 0.06 m represents the cylinder radius. From (7), the torque from the centrifugal force of the impact hammer on the cylinder is calculated as *M*_1_ = 169 N·m.

To ensure that the impact hammer is not spun out before the centrifuge reaches its set pressure, the requirement of *M*_2_ > *M*_1_ should be satisfied. The braking force of electromagnetic caliper-disc brake A (*F*_A_) should satisfy the following requirement:

(8) $$F_{A}>\frac{M_{1}}{r_{\mathrm{disc}}}=846\;N$$

where *r*_2_ = 0.45 m represents the rotational radius of the fully extended impact hammer. The centrifugal force on the impact hammer is calculated as 3627 N and the torque from the centrifugal force of the impact hammer on the cylinder is calculated as *M*_2_ = 218 N·m. Similarly, the braking force of electromagnetic caliper-disc brake B is calculated as 1200 N.

## 5. Simulation Analysis

### 5.1. Simulation Model for Dynamic

**Figure 9:**
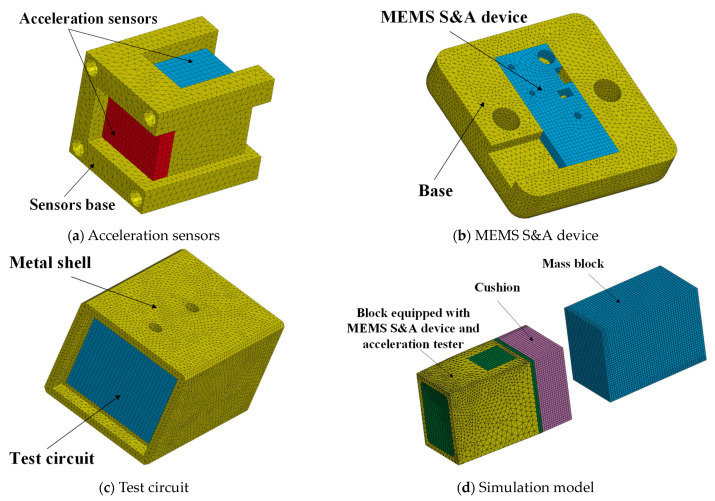
Meshing scheme of the simulation model.

The block mass, equivalent mass of the centrifuge system, and rotational radius of the impact hammer are 0.78 kg, 93.4 kg, and 0.68 m, respectively. A dynamic model of the collision between the impact hammer and block was established using the software ANSYS/LS-DYNA for simulation analysis. SI units (kg, m, s) were adopted for unification. SOLID164 three-dimensional solid elements were used. Adaptive meshing was adopted with a controlled mesh size of 3 mm. The finite-element model after meshing is shown in Figure 9. The material parameters for each component are presented in Table 1.

### 5.2. Effects of Centrifuge Speed on 2D Overload

The centrifuge speed was set as 600, 700, 800, and 900 r/min in the simulation analysis. The initial velocity of the collision corresponding to each centrifuge speed is presented in Table 2.

**Figure 10:**
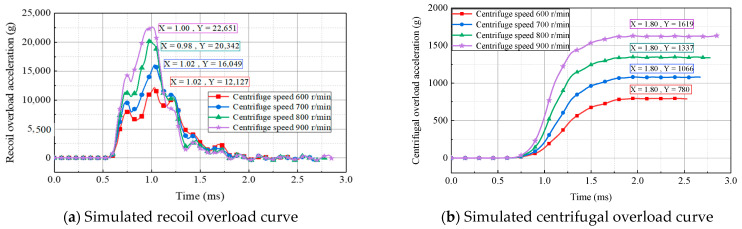
Simulated 2D overload curves for different centrifuge speeds.

The acceleration curves of the simulated recoil overload and centrifugal overload at different centrifuge speeds are presented in Figure 10. As shown, the maximum acceleration for recoil overload and centrifugal overload increased with the centrifuge speed. When the centrifuge speed was 900 r/min, the maximum acceleration for recoil overload and centrifugal overload were 22,651× *g* and 1619× *g*, respectively. The variation in centrifuge speed did not significantly affect the total pulse width of the recoil overload; however, the maximum acceleration of the recoil overload influenced the effective pulse width (the duration of a pulse with an amplitude greater than 10,000× *g*). Since a strong shock can cause the failure of the MEMS S&A device, setting the centrifuge speed at 700 r/min for experimental tests is deemed reasonable. The corresponding acceleration peak for recoil overload was 16,049× *g*, with an effective pulse width of 510 μs, and the corresponding acceleration peak for centrifugal overload was 1066× *g*.

### 5.3. Effects of Cushion Material on 2D Overload

In addition to rubber, nylon, and felt, aluminum foam and epoxy resin are widely used as cushioning materials in engineering practice because of their relatively large elastic moduli. In this section, the aforementioned materials were adopted to make cushions for evaluating the effects of material properties on the overload pulse. The models and parameters of various cushion materials are presented in Table 3.

**Figure 11:**
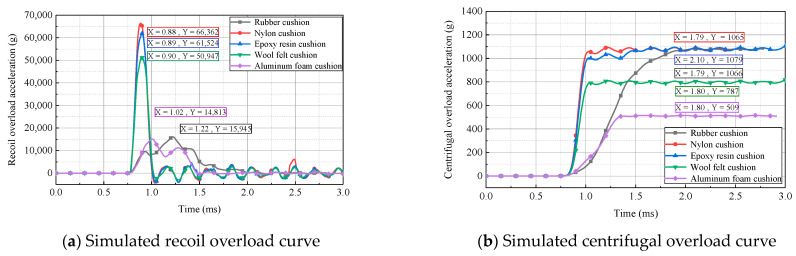
Simulated 2D overload curves for different cushion materials.

The acceleration curves of the simulated recoil overload and centrifugal overload for different cushioning materials at a centrifuge speed of 700 r/min are presented in Figure 11. As shown, compared with nylon, wool felt, and epoxy resin, cushions made of aluminum foam and rubber exhibited superior energy-absorbing and buffering effects, including a longer rising edge of acceleration, a lower peak acceleration, and larger effective pulse width. However, the acceleration peak for centrifugal overload in the case of the aluminum-foam cushion was only 509× *g*, which did not conform to the requirements in the simulation tests. According to an analysis, this was attributed to the fact that significant plastic deformation occurred in the aluminum-foam cushion, causing a considerable energy dissipation and velocity attenuation of the block rotating along the circular track. Rubber cushions can better achieve the conversion between kinetic energy and internal energy, making rubber an ideal cushioning material for simulation tests of 2D overload loading.

### 5.4. Effects of Cushion Shape on 2D Overload

To test the effects of different cushion shapes on the overload pulse, a simulation analysis was performed on rubber cushions with different cross sections and the same thickness and rubber cushions with different thicknesses and the same cross section. The dimensions of the two groups of rubber cushions are presented in Table 4.

**Figure 12:**
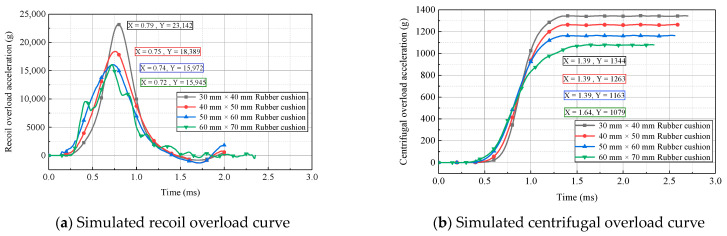
Simulated 2D overload curves for rubber cushions with different cross-sectional areas.

The acceleration curves of the simulated recoil overload and centrifugal overload for cushions with different cross sections and the same thickness at a centrifuge speed of 700 r/min are presented in Figure 12. As shown, the peak accelerations for recoil overload and centrifugal overload decreased with an increase in the cross-sectional area of the rubber cushion. When the rubber-cushion cross-sectional area approached the impact-hammer area (60 mm × 70 mm), the peak acceleration for recoil overload generally became constant. Variations in the cross-sectional area did not significantly affect the total pulse width of the recoil overload, similar to the effects of the centrifuge speed on the overload pulse.

**Figure 13:**
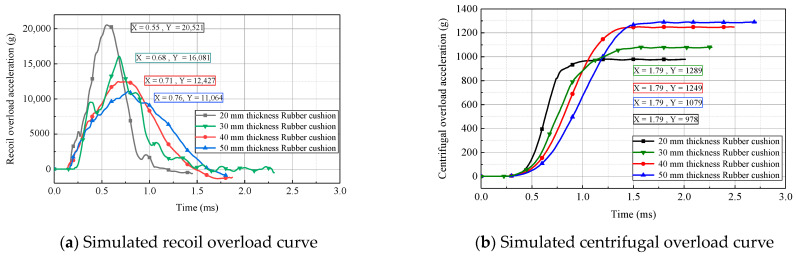
Simulated 2D overload curves for rubber cushions with different thicknesses.

The acceleration curves of the simulated recoil overload and centrifugal overload obtained using cushions with different thicknesses and the same cross-sectional area at a centrifuge speed of 700 r/min are presented in Figure 13. As shown, with an increase in the rubber-cushion thickness, the rising edge of acceleration for recoil overload and centrifugal overload was elongated, while the peak acceleration decreased. The rubber-cushion thickness significantly affected the overload pulse, which exhibited obvious discrepancies among the curves. Therefore, selecting rubber cushions with different thicknesses is an effective method for adjusting the peak and width of the 2D overload pulses.

## 6. Experimental Testing Results

### 6.1. Experimental Testing System Setup

**Figure 14:**
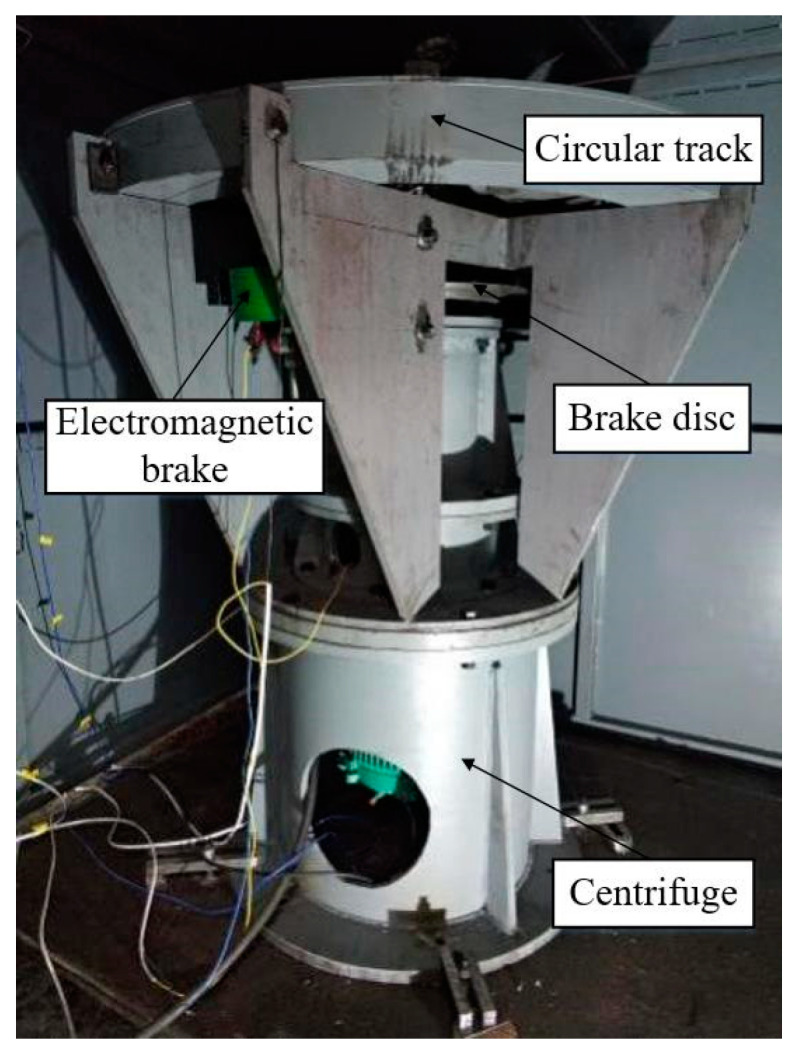
Experimental testing system.

The experimental testing system was developed in this study. A three-phase variable-frequency motor with a controllable speed (FLSMV80M-18.5-1500, Emerson) was adopted for the centrifuge, which had a rated voltage of 380 V, a rated power output of 18.5 kW, and a rated motor speed of 1500 r/min. A frequency converter (M600, Emerson) with a rated voltage of 400 V, rated current of 63 A, rated power output of 30 kW, and working frequency of 50 Hz was used. The motor speed was remotely controlled by the communication between the frequency converter and the upper computer through RS-485. A photograph of the experimental testing system is presented in Figure 14.

**Figure 15:**
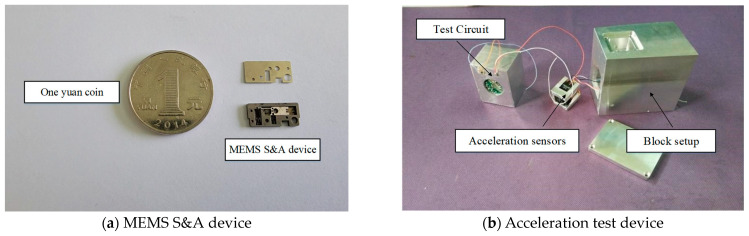
Block setup.

The block setup was composed of three parts: the test piece of the MEMS S&A device, an acceleration tester, and a block shell. Two acceleration sensors for high *g*-values (BM1001) were adopted in the acceleration tester, and their measurement ranges were set as ±30,000× *g* and ±5000× *g*, respectively. After calibration, these two acceleration sensors were installed on sensor bases along the X-axis and Y-axis, which corresponded to the directions of the recoil overload and centrifugal overload, respectively. An internal trigger approach was adopted by the testing system, with a trigger threshold of 1000× *g*, sampling frequency of 200 kHz, and storage time of 16 s. The trigger was manually powered on. To ensure reliable data acquisition and storage, the interior of the test devices was encapsulated with epoxy resin. The photographs of the MEMS S&A device and block setup are shown in Figure 15.

### 6.2. Test Results

**Figure 16:**
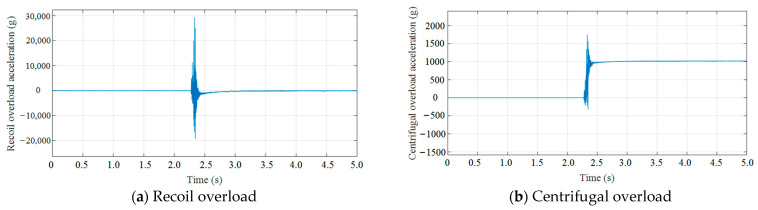
Measured curves of recoil and centrifugal overload.

**Figure 17:**
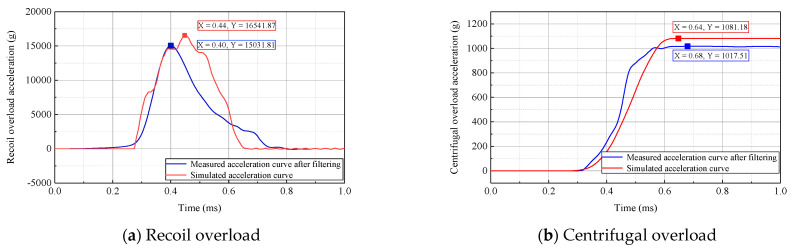
Measured curves of recoil and centrifugal overload after filtering.

In the experimental tests, the centrifuge speed was set to 700 r/min, and a rubber pad with a cross-sectional area of 60 mm × 70 mm and a thickness of 30 mm was selected as the cushion. Data acquired from measurements during the 2D overload simulation test are presented in Figure 16. As shown, the measured acceleration curves contained high-level noise signals; hence, it was necessary to filter the measured curves. MATLAB software was adopted to perform low-pass filtering on the measured data. The 2D overload curves after filtering with a cutoff frequency of 2.5 kHz are presented in Figure 17. As shown, the measured peak acceleration for recoil overload was 15,032× *g*, which was 1510× *g* lower than the simulation curve, with a decrease of 10.05%. The pulse width of the recoil overload could reach 512 μs, which was basically consistent with the simulated curve. The measured peak acceleration for centrifugal overload was 1018× *g*, which was 64× *g* lower than the simulation curve, with a decrease of 6.29%. From the results, this difference is within a reasonable error range. The requirements for 2D overload simulation were satisfied.

**Figure 18:**
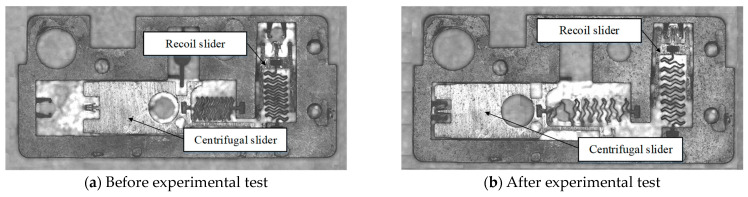
MEMS S&A device status before and after experimental test.

The MEMS S&A device was observed before and after the experimental test using an electron microscope, as shown in Figure 18. The recoil slider head was squeezed into the holder and the recoil slider unit released the constraint on the centrifugal slider unit. The centrifugal slider head was also squeezed into the holder and the MEMS S&A device successfully released the protections.

## 7. Conclusions

An experimental testing system for 2D overload on MEMS S&A devices was designed. A block equipped with a fuze S&A device accelerated instantly upon impact from a centrifuge-driven impact hammer, simulating recoil overload loading. After the collision, the block underwent inertial rotation along a circular track, which is equivalent to the high-speed rotation of projectiles and simulates centrifugal overload loading. The dynamic equations of the experimental testing system and the equations of the impact hammer motions were established. The rotation speed range of the centrifuge was calculated as 600–900 r/min, and the braking forces of electromagnetic brakes A and B were calculated as 900 and 1200 N, respectively. The dynamic model of the experimental testing system was established using the software ANSYS/LS-DYNA, which verified the feasibility of this method. The results indicated that the acceleration magnitude and pulse width for 2D overload can be modified by changing the centrifuge speed and the materials and shapes of the cushions. The developed test system was adopted to perform simulation tests of 2D overload loading on MEMS S&A devices. The acceleration curves for recoil and centrifugal overload were acquired. The measured peak acceleration for recoil overload was 15,032× *g* with an overload pulse width of ~512 μs, whereas the measured acceleration for centrifugal overload was 1018× *g*. The differences between the measured results and the simulation results were within a reasonable error range, thus verifying the effectiveness of the simulation model. The requirements for the 2D overload simulation were satisfied. It should be noted that this article only conducted experimental testing on the MEMS S&A devices of a 20 mm small caliber ammunition fuze (with a centrifugal slider). For some MEMS S&A devices (with two centrifugal sliders), the designed experimental testing system cannot achieve simulation loading. The further work will be to conduct research on MEMS S&A devices for fuzes with a larger caliber and higher overload, and to improve the experimental testing system to meet the reliability performance testing requirements of more types of MEMS S&A devices.

## Figures and Tables

**Table 1:** Model parameters of materials in each component.

Component	Density *ρ*/(kg·m^−3^)	Elastic Modulus *E*/(GPa)	Poisson’s Ratio *ν*	Yield Limit *σ*_s_/GPa
Impact hammer	7850	210	0.28	1.28
Block	2710	69	0.33	0.12
Rubber cushion	1150	1.04	0.42	0.002

**Table 2:** Initial velocity of collision at each centrifuge speed.

No.	Centrifuge Speed/(r/min)	Initial Velocity of Collision/(m/s)
1	600	42.73
2	700	49.85
3	800	56.97
4	900	64.09

**Table 3:** Models and parameters of different cushion materials.

Material	Materical Model	Density *ρ*/(kg·m^−3^)	Elastic Modulus *E*/(GPa)	Poisson’s Ratio *ν*
Rubber	MOONEY-RIVLIN_RUBBER	1150	1.04	0.49
Nylon	PLASTIC_KINEMATIC	1100	2.80	0.40
Wool felt	PLASTIC_KINEMATIC	1140	8.50	0.28
Aluminum foam	CRUSHABLE_FOAM	1100	1.20	0.33
Epoxy resin	PLASTIC_KINEMATIC	1150	3.20	0.40

**Table 4:** Dimensional parameters of rubber cushions with different shapes.

Group	Cross-Sectional Area/(mm^2^)	Thickness/(mm)
Rubber cushions with variable cross-section and constant thickness	30 × 40	30
	40 × 50	30
	50 × 60	30
	60 × 70	30
Rubber cushions with variable thickness and constant cross-section	60 × 70	20
	60 × 70	30
	60 × 70	40
	60 × 70	50

## Data Availability

Not applicable.

## References

[1] Wang W., Bi S., Xiang H., Zhan C., Yuan X. (2017). Trigger control characteristics of fuze-recoil simulation system based on electromagnetic launcher. J. Syst. Eng. Electron..

[2] Sharp A., Andrade J., Ruffini N. (2019). Design for reliability for the high reliability fuze. Reliab. Eng. Syst. Saf..

[3] Kan W., Chu E., Qu P., Ren W., Hu T. (2022). An overview of MEMS S&A device and its application in the micro-detonated system. Int. J. Mod. Phys. B.

[4] Cui M., Huang Y., Li J., Meng M. (2020). Design and Experiment of a Novel High-Impact MEMS Ceramic Sandwich Accelerometer for Multi-Layer Target Penetration. IEEE Access.

[5] Xi Z., Nie W., Cao Y. (2021). An overview on the development of MEMS S&A Device. J. Detect. Control.

[6] Du L., Jia S., Nie W., Wang Q. (2011). Fabrication of fuze micro-electro-mechanical system safety device. Chin. J. Mech. Eng..

[7] Rehan M., Mansoor M. (2021). Application of MEMS in safety and arming devices: An overview. Microsyst. Technol..

[8] Wang K., Hu T., Zhao Y., Ren W., Wang Y. (2023). Design of an intelligent MEMS safety and arming device with a condition feedback function. Micromachines.

[9] Su W., Lou W., Feng H., Zhao Y., He B. (2023). Research on MEMS Solid-State Fuse Logic Control Chip Based on Electrical Explosion Effect. Micromachines.

[10] Hu T., Fang K., Zhang Z., Jiang X., Zhao Y. (2019). The hybrid fabrication process of metal/silicon composite structure for MEMS S&A Device. Micromachines.

[11] Tu H., Sun Z., Qian Y., Liu Q. (2017). Reliability analysis and improvement of MEMS-based safety and arming device in fuze. Acta Armamentarii.

[12] Wang W., Liu W., Zou J., Huo P. (2011). A MEMS safe and arm device for spin stabilized ammunition fuze. Adv. Mater. Res..

[13] Lei S., Cao Y., Nie W., Xi Z., Yao J., Zhu H., Lu H. (2022). Research on mechanical responses of a novel inertially driven MEMS safety and arming device under dual-environment inertial loads. IEEE Sens. J..

[14] Wu Z., Ma T., Zhang Y., Zhang H. (2020). Ground simulation test of 2D dynamic overload environment of fuze launching. Shock Vibr..

[15] Lian J., Li J., Xu L. (2019). The Effect of Displacement Constraints on the Failure of MEMS Tuning Fork Gyroscopes under Shock Impact. Micromachines.

[16] Srikar V., Senturia S. (2002). The reliability of microelectromechanical systems (MEMS) in shock environments. J. Microelectromech. Syst..

[17] Xu K., Zhang W., Hao Y. (2018). Mechanical latching stops for reliability improvement of MEMS in shock environments. Microsyst. Technol..

[18] Zhan C., Xiang H., Lei B. (2015). A study on dynamic simulation of solenoid fuzes. J. Acad. Armored Force Eng..

[19] Qin Y., Shen Y., Zou X., Hao Y. (2022). Simulation and test of a MEMS arming device for a fuze. Micromachines.

[20] Wang P., Sui L., Li G. (2015). A simulation device for loading process of MEMS safety mechanisms. J. Detect. Control.

[21] Xiang H., Lei B., Chen L. (2005). A single-chip-microcomputer-based system for simulating dual environmental forces on fuzes. Ordnance Ind. Autom..

[22] Zhao J., Gao Y. (2003). A study on practical internal ballistics technology of gas gun. J. Taiyuan Univ. Technol..

[23] Zhong W., Zhao X., Qi X. (2005). A study on internal ballistics modeling and launching environment simulation of gas gun. J. Dyn. Control.

[24] Zhang Y., Ma T., Zhang H., Wu Y., Wu Z., Yu J. (2020). Ground simulation tests in two-dimensional dynamic acceleration environment. Appl. Sci..

[25] Zhang Y., Ma T., Sun T. (2016). Simulation method of centrifugal turntable for two-dimensional acceleration overload environment of missiles. J. Proj. Rocket. Missiles Guid..

[26] Zhang Y., Ma T., Zu J. (2012). Vector turntable centrifuge started by air cannon for missile launching simulation. J. Detect. Control.

[27] Yang Q., Qi X., Zhao Z., Li Y., Han M. (2012). Factors for simulating recoil acceleration of fuze based on impact deceleration method. J. Proj. Rocket. Missiles Guid..

[28] Qian H., Gao R., Wang H. (2013). Study on simulation of double environmental forces of liquid reserve battery for fuses. Struct. Environ. Eng..

[29] Xu M., Yu N., Liu J. (2013). The dual-environment testing method of accumulator in fuze. Environ. Eng..

[30] Lin C. (2017). Study of Setback and Spin Environment Simulation of Fuze in Launch Process Based on Electromagnetic Emission Technology. Master’s Thesis.

[31] Zhang J. (2014). Study on Failure Mechanisms and Analysis Methods of MEMS Safety System in Complex Mechanical Environment. Master’s Thesis.

[32] Wu S., Li K., Zhang Z. (2010). Strong Impact Testing and Testing Techniques.

